# Dominant mutations in mtDNA maintenance gene *SSBP1* cause optic atrophy and foveopathy

**DOI:** 10.1172/JCI128513

**Published:** 2019-11-18

**Authors:** Camille Piro-Mégy, Emmanuelle Sarzi, Aleix Tarrés-Solé, Marie Péquignot, Fenna Hensen, Mélanie Quilès, Gaël Manes, Arka Chakraborty, Audrey Sénéchal, Béatrice Bocquet, Chantal Cazevieille, Agathe Roubertie, Agnès Müller, Majida Charif, David Goudenège, Guy Lenaers, Helmut Wilhelm, Ulrich Kellner, Nicole Weisschuh, Bernd Wissinger, Xavier Zanlonghi, Christian Hamel, Johannes N. Spelbrink, Maria Sola, Cécile Delettre

**Affiliations:** 1 Institute of Neurosciences of Montpellier, INSERM, University of Montpellier, Montpellier, France.; 2 Structural MitoLab, Department of Structural Biology, “Maria de Maeztu” Unit of Excellence, Molecular Biology Institute Barcelona (IBMB-CSIC), Barcelona, Spain.; 3 Radboud Center for Mitochondrial Medicine, Department of Paediatrics, Radboudumc, Nijmegen, Netherlands.; 4 CHU Montpellier, Centre of Reference for Genetic Sensory Diseases, Gui de Chauliac Hospital, Montpellier, France.; 5 Faculté de Pharmacie, Université de Montpellier, Montpellier, France.; 6 UMR CNRS 6015-INSERM U1083, MitoVasc Institute, Angers University, Angers, France.; 7 University Eye Hospital, Centre for Ophthalmology, University of Tübingen, Tübingen, Germany.; 8 Rare Retinal Disease Center, AugenZentrum Siegburg, MVZ Augenärztliches Diagnostik- und Therapiecentrum Siegburg GmbH, Siegburg, Germany.; 9 Institute for Ophthalmic Research, Centre for Ophthalmology, University of Tübingen, Tübingen, Germany.; 10 Centre de Compétence Maladie Rares, Clinique Pluridisciplinaire Jules Verne, Nantes, France.

**Keywords:** Genetics, Ophthalmology, Mitochondria, Neurodegeneration

## Abstract

Mutations in genes encoding components of the mitochondrial DNA (mtDNA) replication machinery cause mtDNA depletion syndromes (MDSs), which associate ocular features with severe neurological syndromes. Here, we identified heterozygous missense mutations in single-strand binding protein 1 (*SSBP1*) in 5 unrelated families, leading to the R38Q and R107Q amino acid changes in the mitochondrial single-stranded DNA-binding protein, a crucial protein involved in mtDNA replication. All affected individuals presented optic atrophy, associated with foveopathy in half of the cases. To uncover the structural features underlying SSBP1 mutations, we determined a revised SSBP1 crystal structure. Structural analysis suggested that both mutations affect dimer interactions and presumably distort the DNA-binding region. Using patient fibroblasts, we validated that the R38Q variant destabilizes SSBP1 dimer/tetramer formation, affects mtDNA replication, and induces mtDNA depletion. Our study showing that mutations in *SSBP1* cause a form of dominant optic atrophy frequently accompanied with foveopathy brings insights into mtDNA maintenance disorders.

## Graphical abstract

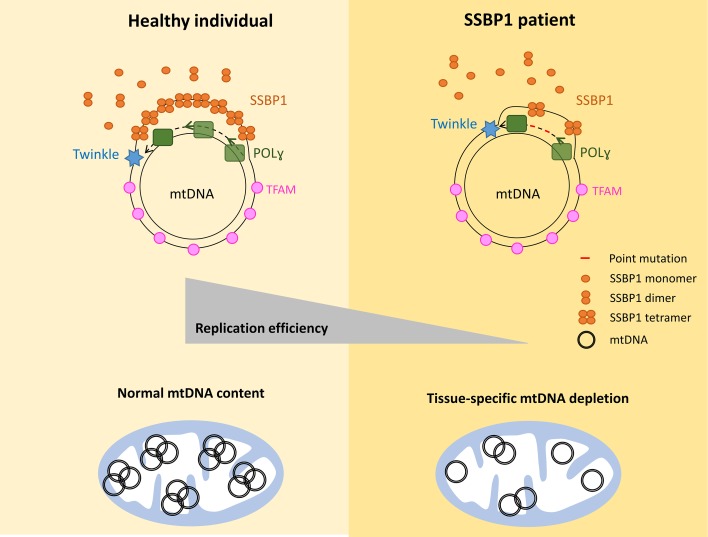

## Introduction

Mitochondrial diseases are clinically heterogeneous and can be caused by mutations in either nuclear or mitochondrial DNA (mtDNA) (1). Mutations in genes known to be essential for mtDNA maintenance are recognized as causing both mtDNA depletion syndromes (MDSs) and mtDNA multiple deletion syndromes (2). Such genes encode components of the mtDNA replication machinery (POLG, POLG2, TWNK, TFAM, RNASEH1, MGME1, and DNA2), dNTP supply for mtDNA synthesis (TK2, DGUOK, SUCLG1, SUCLA2, ABAT, RRM2B, TYMP, SLC25A4, AGK and MPV17), and mitochondrial dynamics (OPA1, MFN2). Ocular involvement is a well-known clinical feature of mitochondrial disease that may occur as retinal, macular, optic nerve dysfunction, or progressive external ophthalmoplegia (PEO) and ptosis involving the extraocular muscles. In MDS and multiple deletion syndromes, ocular features are rarely isolated and may be associated with neurological and/or multisystemic presentation, including muscle, liver, and kidney.

Autosomal dominant optic atrophy (ADOA) is characterized by progressive visual loss, central scotoma, dyschromatopsia, and optic atrophy due to degeneration of the retinal ganglion cells and their axons, which form the optic nerve. Mutations in *OPA1*, which encodes a dynamin-like GTPase involved in mitochondrial fusion and mtDNA maintenance (3–8), are the leading cause of ADOA (9, 10). However, a marked fraction of ADOA manifestations still have unknown origins and are likely caused by mutations in other genes.

Here, we report heterozygous mutations in the mitochondrial single-stranded DNA-binding protein single-strand binding protein 1 (SSBP1) in 5 unrelated families with nonsyndromic dominant optic atrophy that in half of the cases is associated with a striking occurrence of foveopathy. SSBP1 is a key protein for the mtDNA replication machinery. SSBP1 is required to stabilize single-stranded mtDNA (ssmtDNA) and stimulate DNA synthesis by POLG (11). Patient fibroblasts displayed unstable formation of dimers and tetramers of SSBP1 and distorted mtDNA replication, leading to a decrease in mtDNA copy number. We provide evidence that human SSBP1 deficiency engenders an mtDNA syndrome that results in an isolated visual defect.

## Results

### Identification of mutations in SSBP1 in families with dominant optic atrophy.

**Figure 1:**
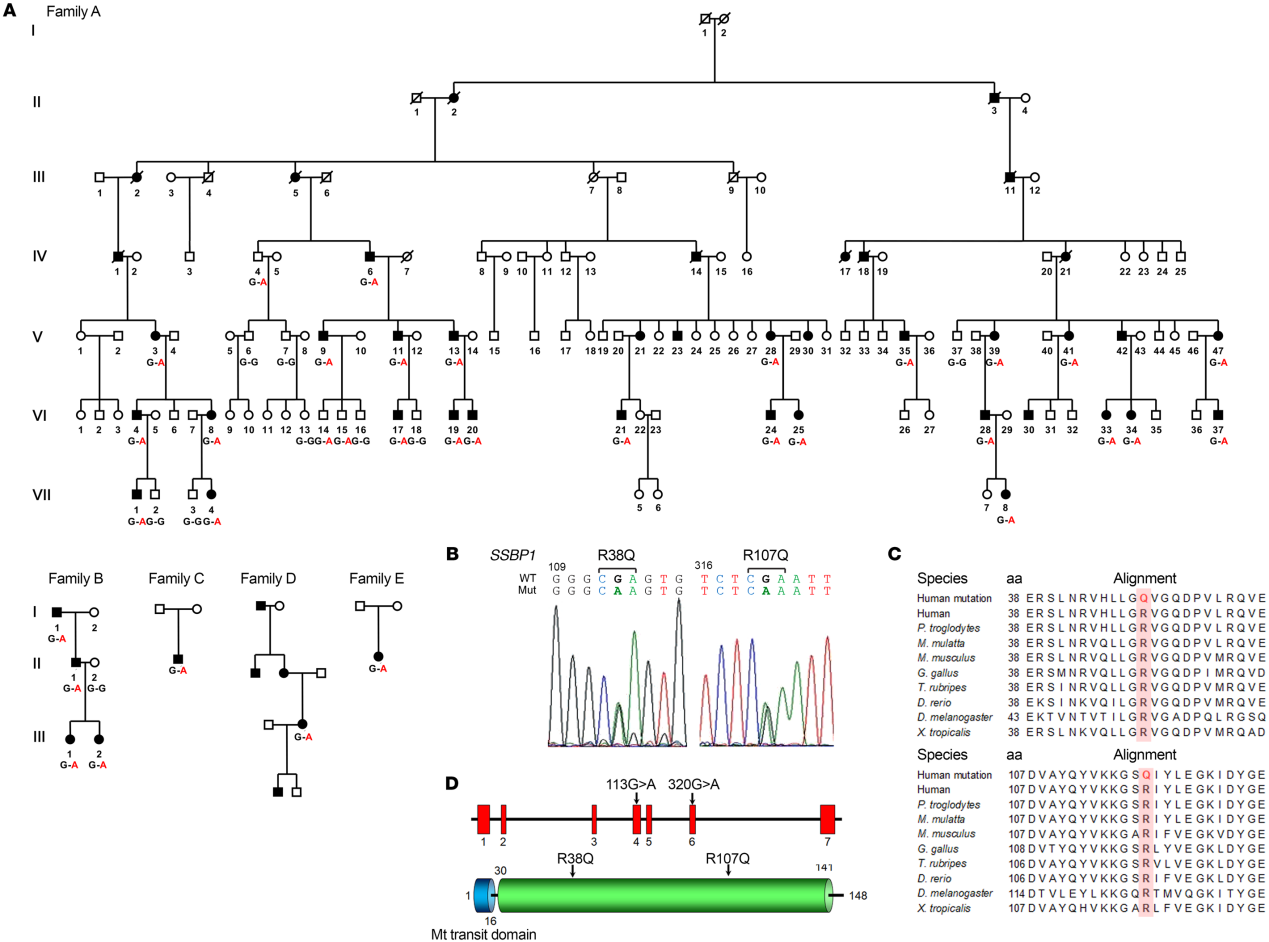
Pedigrees with SSBP1-dominant mutation segregation and localization of the 2 altered residues on SSBP1 gene and protein. (**A**) Pedigree showing males (squares) and females (circles) of the families carrying the pathogenic c.113G>A variant (families A and B) or c.320G>A (families C, D, and E) in the *SSBP1* gene. Black symbols denote affected family members, and white symbols denote unaffected family members. The mutation status of each analyzed family member is indicated. (**B**) Electropherograms of genomic DNA sequencing from family A (left panel) and family D (right panel). WT and mutant (Mut) alleles are indicated. All patients were heterozygous for the identified mutation. (**C**) Sequence alignments showing conservation of the 2 affected amino acid residues between different species. (**D**) Schematic representation of the human SSBP1 (gene on the top, protein on the bottom) with the localization of the 2 mutations. Red squares represent exons, and regions corresponding to the mitochondrial transit domain (blue) and DNA-binding domain (green) are shown.

We performed whole exome sequencing (WES) in one French family (family A) including multiple patients with ADOA to identify the causative gene defect. WES (Human All Exon V5, Agilent followed by Illumina HiSeq2000) was performed on 9 individuals (IV:4, IV:6, V:3, V:9, V:11, VI:4, VI:8, VI:17, and VI:18). Seven were affected (IV:6, V:3, V:9, V:11, VI:4, VI:8, VI:17), one (IV:4) had uncertain phenotype, and one was unaffected (VI:18) (Figure 1A). We confirmed that no mutation in genes previously associated with ADOA or maculopathy was present in these patients. After filtering for variants potentially affecting protein function due to the introduction of missense, nonsense, splicing, or frameshift mutations in coding regions, we focused our analysis on genes that code for mitochondrial proteins. We identified a c.113G>A (p.Arg38Gln) substitution in exon 4 of *SSBP1* (MIM: 600439) (Figure 1, B–D), which encodes the mitochondrial single-stranded DNA-binding protein, a key protein in the regulation of mtDNA replication (12). This variant was not found in the sequenced alleles list from the Genome Aggregation Database (gnomAD). The mutation changes an amino acid residue evolutionarily conserved among vertebrates (Figure 1C) and is predicted to be pathogenic by the Sorting Intolerant From Tolerant (SIFT) and PolyPhen-2 databases. The Combined Annotation Dependent Depletion (CADD) score for this variant was 32, meaning that the variant was predicted to be highly pathogenic. Sanger sequencing demonstrated that the variant segregated with the optic atrophy in all sequenced individuals except for the 2 children VI:14 and VI:15 in family A who so far were clinically unaffected.

Screening of *SSBP1* in a cohort of 174 European probands with inherited optic atrophy and without genetic diagnosis identified 4 additional German families, families B, C, D, and E, with an SSBP1 mutation (Figure 1A). Family B was also heterozygous for the c.113G>A (p.Arg38Gln) mutation in exon 4. Families C, D, and E were heterozygous for a mutation we believe to be novel, c.320G>A, in exon 6, causing a p.Arg107Gln amino acid substitution (Figure 1, B–D). This missense mutation also occurred within a region conserved among species (Figure 1C) and was absent in gnomAD. The CADD score for this variant was 24.3, suggesting that the variant was predicted to be pathogenic. While Arg107Gln was absent in the gnomAD database, another variant at the same codon, Arg107X, was present in 2 alleles from European (non-Finnish) individuals with an allele frequency of 6.37e-5. The 2 mutations were located in different exons of the gene and caused amino acid exchanges 69 residues apart in the polypeptide sequence (Figure 1D).

### Clinical phenotype of patients with SSBP1 mutations.

**Figure 2:**
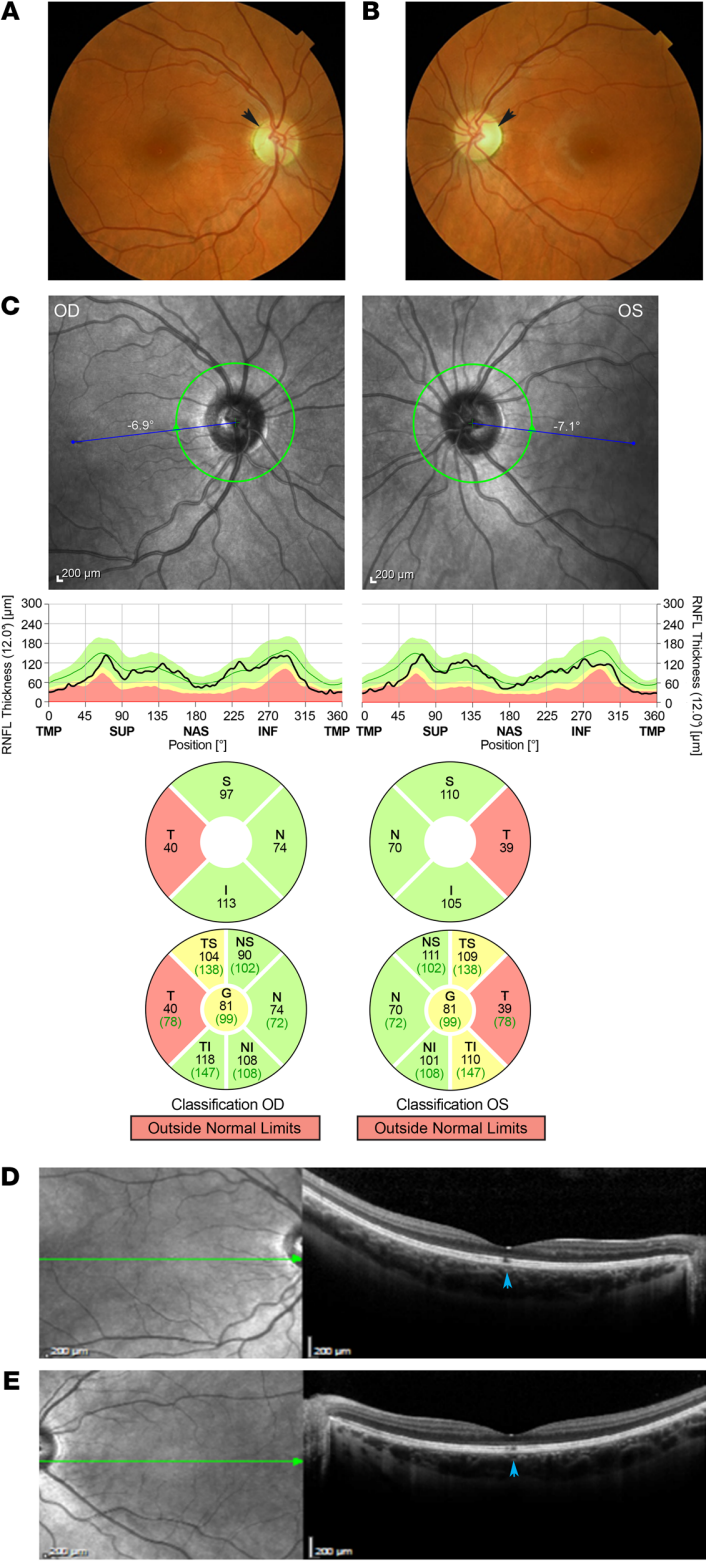
Clinical features of SSBP1 patients. Combined optic atrophy and foveopathy of individual VI-25 from family A. (**A**) Ocular fundus photographs of the right eye and (**B**) left eye. Note the symmetrical temporal optic disk pallor (black arrows). (**C**) Optic SD-OCT and retinal nerve fiber layer (RNFL) deviation map confirm a significant decrease of the temporal RNFL thickness. Temporal part thickness is outside normal limits in red. The green area corresponds to the 5th–95th percentiles, the yellow area corresponds to the 1st–5th percentiles, and the red area corresponds to below the 1st percentile. OD, right eye; OS, left eye. (**D**) SD-OCT macular analysis of the right eye and (**E**) of the left eye. SD-OCT reveals a combined foveopathy unsuspected on color photographs. A tiny disruption of both ellipsoid and interdigitation lines is observed in both eyes (blue arrows) beneath the fovea.

The predominant clinical symptom exhibited by the present cohort of patients with *SSBP1* mutations is an optic atrophy. We collected DNA samples from 36 affected and unaffected family members from 4 generations in family A (Table 1) and showed that 28 patients carried the c.113G>A (p.Arg38Glu) mutation in exon 4 of the *SSBP1* gene and 8 family members did not carry the mutation. Of the 28 affected members, 21 patients had complete medical files (Table 1). The remaining 7 patients (IV:4, V:35, V:47, VI:28, VI:33, VI:34, and VI:37) were described as affected using the family history interview. Two juvenile patients (VI-14 and VI-15) sharing the mutation c.113G>A (p.Arg38Glu) were so far asymptomatic, with 20/20 visual acuity, but with some color vision anomalies. All of the 19 symptomatic patients with available medical records had an optic atrophy with a bilateral pallor of the temporal neuroretinal rim (Figure 2, A and B). Visual acuity varied from 20/400 to 20/20. Protan or deutan color defects were noted. Central, coecocentral, and paracentral scotomas with preserved peripheral isopters were identified in all symptomatic patients. Among these 19 patients, 12 also had a foveopathy only discovered by spectral-domain optical coherence tomography (SD-OCT), with tiny bilateral small defects of the ellipsoid zone (EZ) and interdigitation zone (IZ) restricted to the foveola (Figure 2, C–E). The 4 other families exhibited isolated optic atrophy, except for family B, in which the 2 sisters (III:1 and III:2) of the last generation had abnormal fovea.

### SSBP1 expression.

**Figure 3:**
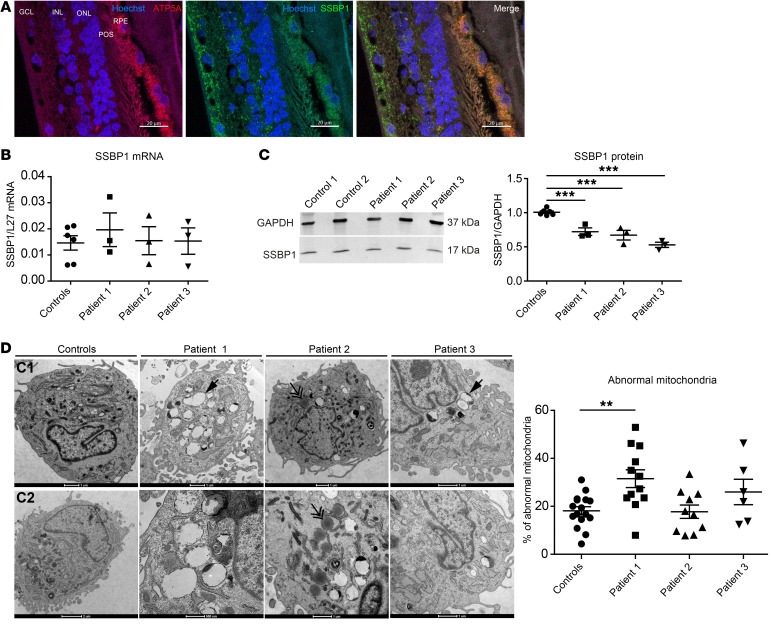
SSBP1 protein expression in human retina and fibroblasts. (**A**) SSBP1 protein expression in human retina. Immunofluorescence labeling was done on retinal cross-sections from healthy human donor. Hoechst was added to label nuclei (blue). SSBP1 localization was done using a specific antibody (green). An antibody against the ATP synthase subunit 5A was used to target mitochondria (red). RPE, retinal pigment epithelium; POS, photoreceptor outer segment; ONL, outer nuclear layer; INL, inner nuclear layer; GCL, ganglion cell layer. Scale bars: 20 μm. (**B**) Quantification of SSBP1 transcript levels in cultured skin fibroblasts from both controls and affected individuals (patient 1, patient 2, and patient 3). mRNA levels were normalized to the reference gene *L27*. (**C**) Western blot in lysates from controls (control 1 and control 2) and patient fibroblasts and densitometric analysis of SSBP1 protein abundance in lysates from both controls and patient fibroblasts. GAPDH was used as a loading control. Data are shown as mean ± SEM. ***P < 0.001. (**D**) Representative ultrastructure of the mitochondria from both controls (C1, C2) and patient fibroblasts by TEM. Scale bar: 1 μm. Single arrows show abnormal mitochondria. Double arrows show lipid droplets. Quantification analyses of abnormal mitochondria (large vacuoles, disturbed cristae) in fibroblasts from both controls and patients. Data are represented as mean percentage of abnormal mitochondria ± SEM in total examined mitochondria. **P < 0.01. All data are representative of 3 independent experiments. One-way ANOVA with Dunnett’s correction was used.

To study the cellular localization of SSBP1 in human retina, human retinal slices were costained with mitochondrial marker of complex V, ATP synthase, and SSBP1. SSBP1 was preferentially expressed in retinal ganglion cells, photoreceptors, and pigmented epithelium (Figure 3A).

To further investigate the molecular consequences of SSBP1 mutations, we characterized skin fibroblasts from 3 affected patients (V:11, VI:17, V:9, respectively known as patient 1, patient 2, patient 3) and 2 healthy control family members (V:14, VI:18, respectively known as control 1, control 2). SSBP1 transcript levels were first examined. No significant differences in the mRNA expression of *SSBP1* were found in patients compared with expression in control fibroblasts (Figure 3B). Western blot analysis showed that SSBP1 was markedly reduced in patients 1, 2, and 3 compared with controls (Figure 3C), indicating that this mutation leads to severe reduction of SSBP1 protein stability. We then determined whether mitochondrial morphology was altered in SSBP1 mutant cells. The mitochondrial network appeared unchanged (Supplemental Figure 1; supplemental material available online with this article; https://doi.org/10.1172/JCI128513DS1), but ultrastructural examination of mitochondria with transmission electron microscopy (TEM) indicated that the overall structure was abnormal in the most affected individual, patient 1, showing swelling and disorganized cristae (Figure 3D). These alterations have been previously noted in SSBP1 knockdown cells (13).

### Revised SSBP1 crystal structure and structural effects of SSBP1 mutations.

**Figure 4:**
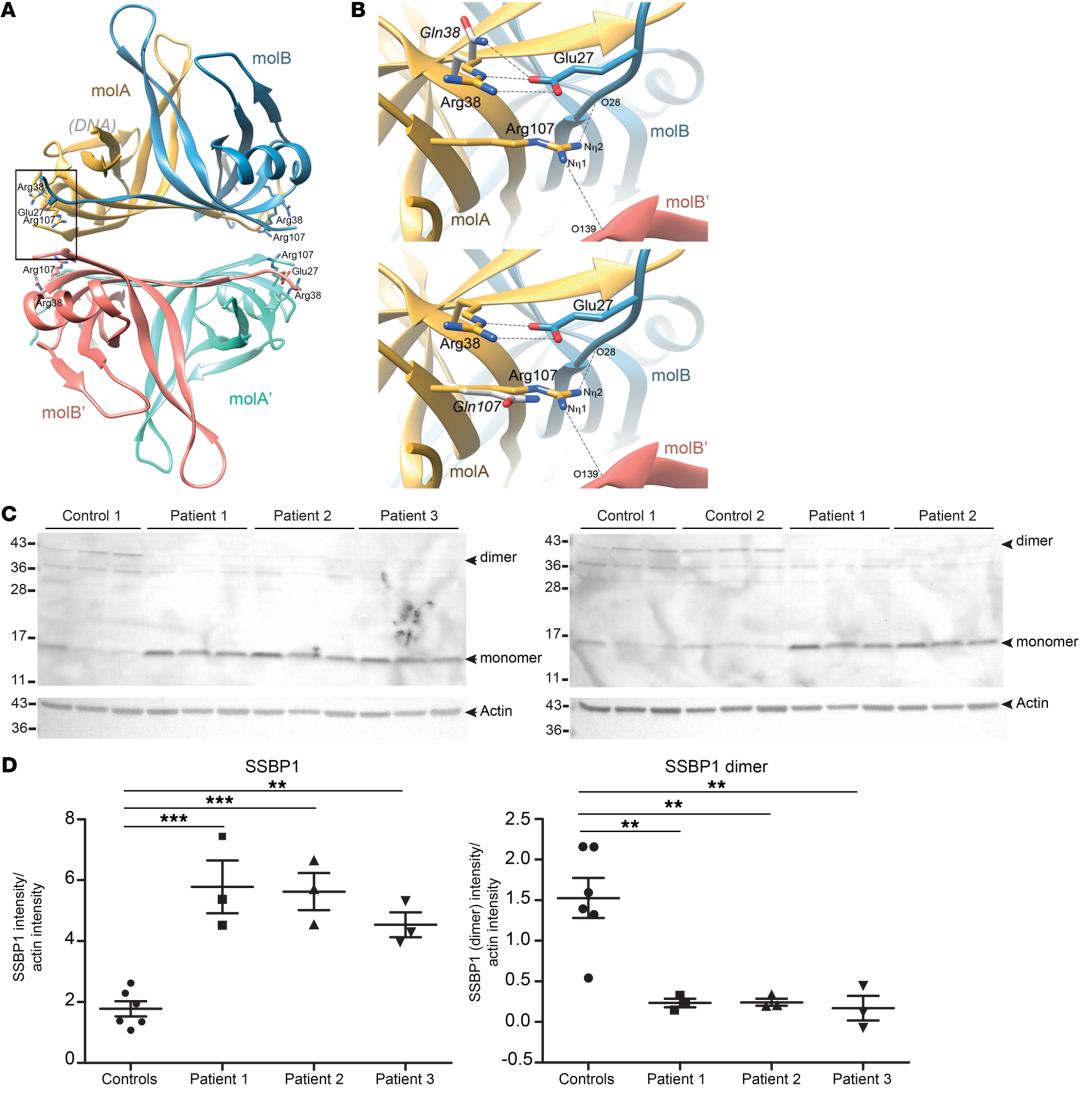
Structural effects of SSBP1 mutations. (**A**) Crystal structure of SSBP1 showing that human SSBP1 consists of a dimer (molecules molA and molB) that contacts a second, symmetrically related dimer (molA′ and molB′), giving rise to the tetrameric biological unit. Both Arg38 and Arg107 are displayed in yellow (frame). (**B**) Zoom-in of mutated residues; Arg38 is replaced by Gln38 (top). The bottom panel is the same, except Arg107 is replaced by Gln 107. In each case, the mutated residue is shown in light gray. (**C**) SDS/PAGE analysis of SSBP1 monomers and dimers in control (control 1 and control 2) and patient fibroblasts (patient 1, patient 2, patient 3). Actin was used as a loading control. For each cell line, 3 independent biological replicates were loaded. (**D**) Densitometric quantification of SSBP1 monomer (left) and dimer (right) in pooled controls and patient fibroblasts. Data are shown as scatter plots with mean ± SEM indicated. **P < 0.01; ***P < 0.001, 1-way ANOVA with Dunnett’s correction. Note that some of the same samples (for control 1, patient 1, and patient 2) were run on both membranes and these thus represent technical replicates. The ratios with actin from both membranes were averaged prior to statistical analysis.

The effects of Arg38Gln and Arg107Gln mutations were elucidated within the 3D structure of SSBP1. In the Protein Data Bank, 3 SSBP1 crystal structures are available (PDB codes 3ULL and 1S3O, refs. 14, 15; and 2DUD, without associated citation). However, these structures show different sequence assignments for the 15 amino acids at the C terminus. To clarify this issue, we solved the crystal structure of SSBP1 at a higher resolution and calculated the electron density maps. Human SSBP1 consists of a dimer (molecules molA and B) that contacts a second, symmetrically related dimer (molA′ and B′), giving rise to the tetrameric biological unit (refs. 15, 16, and Figure 4A).

Strikingly, despite being far apart in the sequence, Arg38 and Arg107 contact each other directly at the end of neighboring β strands of the barrel in a monomer (frame in Figure 4, A and B). Arg38 is close to the surface that (in the homologues) binds the DNA (Figure 4A). Importantly, the guanidinium nitrogens at the end of the Arg38 side chain interact with the side-chain Glu27 carboxylate group from molB (Figure 4B). Thus, Arg38 participates in intermolecular contacts within the dimer (the same contact occurs between Arg38 molB and Glu27 molA). Arg107 is packed against a hydrophobic pocket at the dimer interface, where it interacts with main-chain carbonyls of both molB Arg28 (intradimer contact) and molB′ Phe139 (interdimer contact) (Figure 4B). Therefore, both arginines participate in AB (and BA) dimer interactions, whereas Arg107 additionally contacts the other dimer within the tetramer.

To understand the effect of the mutations, we generated crystal structure–based mutant models employing single-site mutation modeling (20 models per mutant). As a control, we “mutated” the 2 arginines 38 and 107 to arginine (R38R and R107R, respectively) (Figure 4B). The models of these latter showed highly variable orientations for the Arg38 side chain, which sometimes entailed breaking its interaction with Glu27 of molB and often reaching the region plausibly contacted by ssDNA. In contrast, the R38Q mutant showed that the much shorter Gln side chain did not reach the putative DNA-binding region (Figure 4B). Likewise, the substitution of Arg107 for the much shorter Gln increased the distance to the main-chain carbonyls of both Arg28 molB and Phe139-B′ molB′; thus the contacts with the other subunits were lost (Figure 4B). It is worth noting that the stabilizing van der Waals interactions between Arg28 and Arg107 side chains observed in the crystal could not take place in the mutants due to shortening of side chains and the presence of charges at positions were methylenes are found in the WT. This predicts that mutation R38Q has a structural effect on the Arg107 neighbor, which is now less constrained by the mutated, shorter side chain. Therefore, in the R38Q mutant, not only the interaction with molB Glu27 is disrupted but, additionally, the contacts performed by the more free Arg107 might also be destabilized, since this latter can explore more conformations.

Human SSBP1 tetramers have DNA-binding activities. The aforementioned modeling of disease mutations based on the new crystal structure suggested instability of dimer formation. We assessed the effect of R38Q mutation on SSBP1 multimer (dimer, tetramer) stability in patient fibroblasts using gel electrophoresis. Under mild reducing conditions with SDS/PAGE, SSBP1 from patient fibroblasts contained an important increase in monomers with respect to dimers, whereas in controls, dimers were more prominent than monomers (Figure 4, C and D). These findings suggested that the Arg38Gln mutation is sufficient to destabilize the SSBP1 tetramers.

### SSBP1 mutation affects mtDNA replication.

**Figure 5:**
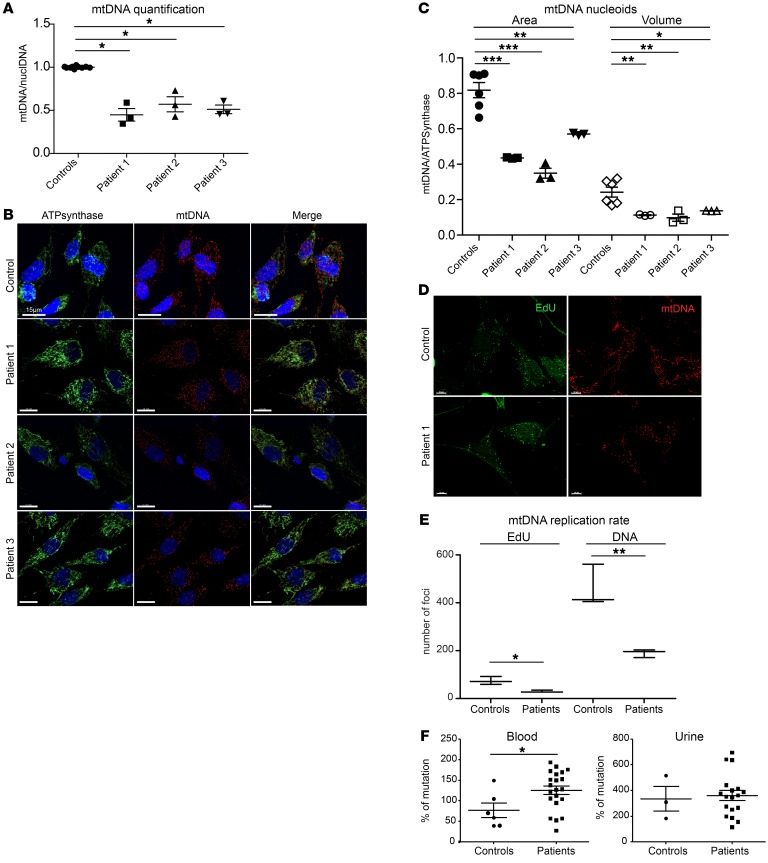
Functional effect of SSBP1 mutation on mtDNA replication. (**A**) Quantification of mtDNA. mtDNA content was measured by qPCR and normalized to a nuclear gene (β-hemoglobin) in both controls and patient fibroblasts (patient 1, patient 2, patient 3). (**B**) Representative confocal imaging of mitochondria using ATPsynthase antibody (green) and mitochondrial nucleoids visualized by DNA antibody (mtDNA, red) in control and patient fibroblasts. Scale bars: 15 μm. (**C**) Quantification of area and volume of mtDNA nucleoids in pooled controls and patient fibroblasts. (**D**) Assessment of mtDNA replication efficiency on cultured skin fibroblasts from control and patient 1. EdU labeling was used to detect and quantify mtDNA synthesis, while anti-DNA antibody was used to quantify all visible mtDNA foci. EdU and total mtDNA foci were counted in both controls and the 3 patient cell lines and pooled for statistical analysis. Scale bars: 10 μm (**E**) Quantification of mtDNA replication rate in controls and patients. Data are shown as mean ± SEM. *P < 0.05; **P < 0.01; **P < 0.001 unpaired *t* test. (**F**) Mutation rates in mtDNA from control and patient blood (left) and urine (right). All data are representative of 3 independent experiments. Mann-Whitney *U* test was used.

To clarify the molecular consequences of SSBP1 mutations, we analyzed patient fibroblasts to detect eventual mtDNA deletion and depletion. mtDNA copy number analysis by quantitative PCR (qPCR) detected significant depletion of mtDNA in all patient fibroblasts tested (Figure 5A). This was also observed by staining the mitochondrial nucleoids with an anti-DNA antibody (Figure 5B). In line with these findings, we observed that TFAM and POLG1 protein levels were also reduced in patient fibroblasts (Supplemental Figure 2). We further evaluated area and volume of nucleoids in patient fibroblasts, disclosing a 75% reduction of anti-DNA immunofluorescence in patients compared with controls, confirming the depletion observed by qPCR and a marked decrease in the number of nucleoids in patient cells (Figure 5C). To further examine the consequence of SSBP1 mutation on mtDNA replication, we visualized newly synthesized mtDNA using EdU incorporation, a marker for DNA synthesis. EdU incorporation was reduced in patient fibroblasts compared with controls (Figure 5, D and E, and Supplemental Figure 3). These results clearly indicate perturbed mtDNA replication, similar to that observed in patients with pathogenic mutations in genes coding for proteins that function directly at the replication fork (POLG, TWINKLE) (17). Since SSBP1 stimulates mtDNA replication, destabilization of the replication machinery may cause mutations and promote mispairing, thereby inducing deletions. To test this eventuality, we collected urine from affected and unaffected individuals from family A (*n* = 29) and sequenced their mtDNA by NGS, together with mtDNA from the blood samples (*n* = 35). After eliminating variants related to the haplogroup differences, analyses of the sequences revealed a significantly higher number of mutations in the mtDNA from the blood samples from the *SSBP1* patients compared with that of controls, while no differences were found between the urine samples (Figure 5F), although the levels of mtDNA mutations were 4 times higher in the mtDNA from urine than in blood. For each mutation, the level of heteroplasmy was inferred by dividing the number of mutated reads by the total number of reads covering the mutated position and distributed according to their frequency in 3 categories: 0%–5%, 5%–10%, and greater than 10%. Although, statistical analyzes never reached a significant *P* value, a trend toward a difference was found for each heteroplasmy level category in blood samples between controls and patients, while not in urine (Supplemental Figures 4 and 5). We then searched for differences between the frequency of mtDNA deletions in blood and urine samplings using eKLIPSE software (18), but without evidence of significant differences. Long-range PCR revealed the absence of mtDNA deletion in the skin biopsies (Supplemental Figure 6). These results suggested that SSBP1 R38Q mutation affects the fidelity of mtDNA replication, but does not promote mtDNA deletion formation in the examined tissue samples.

We then evaluated the effect of the SSBP1 R38Q mutation on mitochondrial respiration using the Agilent Seahorse XF Analyzer. Even when the patient’s values were somewhat lower than those of controls, basal and maximal respiration were not significantly different between the controls and patient fibroblasts (Supplemental Figure 7). Complex I– and complex II–driven respiration remained unchanged between controls and patient fibroblasts. Protein levels were quantified for each complex, and the protein abundance of complex I (GRIM19), complex II (SDHA), complex III (UQCRC2), and complex V (ATP synthase) subunits did not differ between control and patient cells (Supplemental Figure 8). Complex IV (MTCO1) protein levels were slightly decreased only in patient 1 compared with controls.

## Discussion

With the exception of *SSBP1*, a key component of the mitochondrial replication fork (19), to date, all nuclear genes directly involved in mtDNA replication (*POLG*, *POLG2*, *TWINKLE*, *RNASEH1*, *DNA2*, *MGME1*) have been associated with mitochondrial disease, mtDNA depletion, and/or mtDNA deletion (2). Nevertheless, recent findings showed that the combination of a heterozygous *SSBP1* variant with an m.1555A>G mtDNA variant may be responsible for maternally inherited deafness (20).

In this work, we identified *SSBP1* as a gene associated with tissue-specific mtDNA depletion diseases and with an unexpected dominant phenotype: an autosomal dominant nonsyndromic optic atrophy with foveopathy. Using WES on DNA from 8 affected members from a large family with clinical features of ADOA and subsequent targeted sequencing of the candidate gene in additional ADOA families, we identified for what we believe is the first time mutations in *SSBP1* in 5 independent families with ADOA, thereby expanding the number of genes associated with ADOA. The patients have an optic atrophy with a clinical presentation similar to *OPA1* mutations, but also a specific clinical feature that consists of a singular foveopathy never, to our knowledge, described in any mitochondriopathy or optic neuropathy. Nucleoside analog reverse transcriptase inhibitors (NRTIs) are known to be toxic for mitochondria, acting by principally inhibiting mtDNA polymerase γ and leading to mtDNA depletion (21). Interestingly, foveopathy alone as well as foveopathy with optic neuropathy, have been encountered in HIV patients in consequence of antiretroviral treatment (22, 23). These data support our findings and suggest a pathophysiological link between mtDNA replication maintenance and retinal/optic nerve disease. Dominant pathogenic mutations in genes involved in mtDNA replication usually cause autosomal-dominant progressive external ophtalmoplegia (adPEO), rapidly followed by extraocular symptoms and associated with multiple mtDNA deletions (24–26). Nevertheless, rare cases of optic atrophy have been attributed to POLG mutations (27). On the other hand, *OPA1*, the major gene for autosomal ADOA, has been previously reported in adPEO (6).

*SSBP1* encodes a mitochondrial ssDNA-binding protein and interacts with POLG and TWINKLE at the mitochondrial replication fork to stimulate the synthesis of mtDNA (28). During replication, SSBP1 binds to ssmtDNA as a tetramer and prevents reannealing and nucleolytic attacks. The first crystal structure of human SSBP1 suggested that ssmtDNA forms a coil around the tetramer guided by a malleable loop (15). Our molecular modeling on our new, higher resolution crystal structure of SSBP1 suggests that R38Q and R107Q substitutions may affect both multimerization of SSBP1 and DNA binding. In particular, the structure shows that both Arg38 and Arg107 interact with each other and with amino acid residues from the other subunit in the AB dimer. Arg107, being closer to the dimer-dimer interface, further performs interactions with the B′ molecule of the other dimer and therefore is expected to further stabilize the tetramer. In addition, Arg38 might be involved in DNA binding, as its long side chain reaches the DNA-binding surface found in homologous protein structures. The mutated Arg38Gln shorter side chain is unable to reach this region. A further effect of each of these single-site mutations is on the remaining nonmutated arginine. Since the mutations cancel the van der Waals interaction between arginines 38 and 107, the stabilizing effect of this contact is lost; thus one mutation affects both amino acids. Finally, our calculations show that the arginine side chains are flexible and that the electrostatic interactions of the terminal amide groups are reversible. This further suggests that the flexibility of the arginine long side chain allows maintaining such interactions despite local conformational changes. Mutations to shorter glutamines also cancel these properties. Using patient fibroblasts, we validated that the R38Q mutation destabilizes dimer formation of SSBP1, likely also affecting tetramer formation, affects mtDNA replication, and induces a moderate mtDNA depletion. The effect of mutations in SSBP1 on mtDNA replication could be due to alterations in the stimulation of TWINKLE and/or POLG slowing down the replication and causing mtDNA depletion. Because the mutant protein is dominantly inherited, it likely competes with the WT in forming dimers and tetramers, leading to either a gain-of-function or a dominant negative effect on SSBP1 function. The R107X reported in the genomAD database will probably result in reduced SSBP1 levels due to mRNA decay or protein instability and may have otherwise no effect or no phenotype. If the R107X is stable, it may be unlikely for it to be incorporated in a multimer, in which case it will not be dominant negative.

Autosomal dominant mutations impairing the mitochondrial replication fork have been described as causing frequent stalling of the replication machinery (29, 30). mtDNA depletion in patient fibroblasts could be explained by an increase pausing of the replication fork. Total loss of mtDNA has been described in a model of deletion of the yeast *SSBP1* homologue *RIM1* (31). Moreover, knockdown of the Drosophila SSBP1 resulted in severe depletion of mtDNA in S2 cells (32). We also determined the mtDNA point mutation load in blood and urine samples and found increased numbers of mtDNA point mutations in blood samples from patients. However, we did not see mitochondrial deletions in patient blood, urine, and fibroblasts, a classic hallmark of MDS. The absence of detectable mtDNA deletions in fibroblasts is well known in mtDNA maintenance diseases (33) and is probably due to a faster loss of mtDNA deletions in cells with rapid turnover. Unexpectedly, patient fibroblasts were able to maintain normal mitochondrial respiration, indicating that the reduced mtDNA levels are still sufficient for normal mitochondrial physiology.

In summary, we have identified missense mutations in *SSBP1* as a cause of ADOA that is combined with a singular foveopathy. Our study shows that the defect in SSBP1 alters mtDNA replication and induces a moderate mtDNA depletion in fibroblasts. Our findings extend the list of genes that can cause optic atrophy when mutated and support the profound importance of this protein in visual function. We also show that dominant defects in proteins involved in mtDNA replication are candidates for optic atrophies. Understanding why proteins involved in the same pathway, such as mtDNA replication, contribute to different clinical manifestations remains challenging. In our study, optic atrophy is clearly unexpected for mutations in *SSBP1*, an mtDNA maintenance–related gene. However, *OPA1*, the major gene responsible for ADOA, is also involved in the maintenance of mitochondrial genome integrity, albeit more indirectly.

Our study increases the range of clinical presentations associated with mutations in genes involved in mtDNA replication, underlining particular clinical signs such as dominant optic atrophy and foveopathy. Moreover, our results add SSBP1 to the increasing list of dominant optic atrophy–causing genes.

## Methods

### Patients.

Age of onset, initial symptoms, best-corrected visual acuity, Goldmann visual fields, and multimodal imaging (using NIDEK non-mydriatic camera and Heidelberg Spectralis OCT device, full-fied [Ff-ERG] and multifocal electroretinography [Mf-ERG]) were reviewed in affected members of families.

### Targeted exome sequencing.

Genomic DNA was captured using the Agilent enrichment solution method with their biotinylated oligonucleotide bank probes (Human All Exon V5, Agilent). The paired-end high-throughput sequencing was performed using Illumina HiSeq 2000. For a detailed explanation of the protocol, see the publication in *Nature Methods* (34). Sequence capture, enrichment, and elution were performed according to the supplier’s protocol and recommendations (SureSelect, Agilent) without modification. Briefly, 3 μg of each genomic DNA was fragmented by sonication and purified to obtain fragments of 150–200 bp. The oligonucleotide adapters for sequencing both ends of the fragments were ligated and repaired with adenine, added to the ends, and then purified and enriched by 4 to 6 PCR cycles. Then, 500 ng of purified libraries were hybridized to a SureSelect capture oligonucleotides bank for 24 hours. After hybridization, washing, and elution, the eluted fraction was amplified by 10 to 12 PCR cycles and purified and quantified by qPCR to obtain sufficient DNA template for subsequent downstream processes. Each DNA sample was eluted and enriched, then sequenced on an Illumina HiSeq 2000 for 75 bp sequences from each end. Image analysis and determination of the bases were done using Illumina RTA software, version 1.14, with default settings. Sequencing data were deposited in the NCBI’s Sequence Read Archive (SRA BioProject PRJNA562561). BioSample metadata were deposited in the NCBI’s BioSample database (BioSample SAMN12644719, SAMN12644720, SAMN12644721, SAMN12644722, SAMN12644723, SAMN12644724, SAMN12644725, SAMN12644726, SAMN12644727).

### Bioinformatics analysis.

Bioinformatics analysis of sequenced data was based on the Illumina CASAVA1.8 pipeline. CASAVA1.8 is a suite of scripts including the sequence alignment to the complete genome (build37 for human) and counting and detection of allelic variants (SNPs and indels). The alignment algorithm used was ELANDv2e (Maloney alignment and multi-seed reducing artifactual mismatches). Note that only the positions included in the coordinates of the target regions were preserved.

Annotation of genetic variation was carried out internally, including gene annotation (RefSeq and Ensembl) and referenced polymorphisms (dbsnp132, 1000Genomes), followed by a characterization of the mutation (exon, intron, silent, false nonsense, etc.). For each position, exomic frequencies were also determined (Homo and HTZ), taking into account at least 150 exomes sequenced in IntegraGen. The results were obtained per sample, as tabulated text files. We also provided the control quality results of sequencing targets (depth/coverage).

### Cell cultures.

Fibroblasts were cultured from skin biopsies taken after obtaining informed consent from 2 controls and 3 affected patients carrying mutations in the *SSBP1* gene (p.R38Q). Fibroblasts were cultured in two-thirds of RPMI 1640 Medium (Thermo Fisher Scientific) supplemented with 10% FBS, 1% penicillin–streptomycin–amphotericin B (Thermo Fisher Scientific), and one-third of AmnioMax-C100 basal media (Thermo Fisher Scientific*)* with Amniomax C100 supplement (Thermo Fisher Scientific).

### Reverse transcription and qPCR studies.

Reverse-transcription qPCR (RT-qPCR) was used to analyze the expression of SSBP1 mRNA in fibroblasts. RNA was isolated using the QiaShredder and RNeasy Mini Kits (QIAGEN) according to the manufacturer’s instructions. RNA (1 μg) was reverse transcribed using the SuperScript III First Strand Synthesis System Kit (Invitrogen). qPCR amplification was performed using SSBP1-specific primers (forward: TCAGGACCCTGTCTTGAGA; reverse: GATATTCTGTGCCATGTTGTC) and Light Cycler 480 SYBR Green I Master Mix on a Light Cycler 480 II thermal cycler (Roche). Results were normalized to ribosomal protein *L27* gene expression and were analyzed using LightCyclerVR 480 software and the Microsoft Excel program (*n* = 3 independent experiments).

### mtDNA measurements.

Total DNA purifications were performed using the DNeasy Kit (QIAGEN) and quantified by spectrophotometry (Nano-Drop 1000). Nuclear and mtDNA respective abundances were quantified in triplicate by qPCR under standard conditions using the LightCycler FastStart DNA Master SYBR Green I Kit (Roche), with the human primers HunuclS (ACACAACTGTGTTCACTAGC), HunuclAS (CCAACTTCATCCACGTTCA), HumitS (TTCAGACCGGAGTAATCCAG), and HumitAS (AGTAGAACAGCGATGGTGAG). The ratios between mtDNA and nuclear DNA concentrations were reported on graphs(*n* = 3 independent experiments).

### Mitochondrial respiration measurements.

Controls and patients’ fibroblasts were seeded at 7000 cells/well in 100 μl of medium (RPMI/Amniomax) in Seahorse XF96 Cell Culture Microplates coated with 1/100 dilution Corning Matrigel hESC-qualified matrix (Dominique Dutscher) and using 8 replicates. Cells were incubated for 48 hours at 37°C in a 5% CO_2_ atmosphere.

Cellular oxygen consumption (oxygen consumption rate [OCR]) was assayed using the Seahorse XF96 Extracellular Flux Analyser with sequential addition of oligomycin (0.5 μM), carbonyl cyanide 4-(trifluoromethoxy)phenylhydrazone (FCCP, 1 μM), rotenone (1 μM), and antimycin (0.05 μM) to measure basal and maximal respiration. OCR was normalized to protein content. For mitochondrial complexes I and II, respiratory rates were measured as previously described (35).

### TEM.

Cells were immersed in a solution of 2.5% glutaraldehyde in PHEM buffer (1×, pH 7.4) overnight at 4°C, rinsed in PHEM buffer, and post-fixed in a 0.5% osmic acid for 2 hours in the dark and at room temperature. After 2 rinses in PHEM buffer, cells were dehydrated in a graded series of ethanol solutions (30%–100%) and were embedded in EmBed 812 using an Automated Microwave Tissue Processor for Electronic Microscopy (Leica). Ultrathin sections (70 nm; Leica-Reichert Ultracut E) were collected at different levels of each block. These sections were stained with uranyl acetate and lead citrate before examination in a Tecnai F20 TEM at 200KV in the Montpellier Rio Imaging (MRI) and Electron Microscopy Platform (COMET) facilities at the Institute of Neurosciences of Montpellier. To evaluate the percentages of abnormal mitochondria in the different fibroblasts, we counted the number of mitochondria (247 in control C1, 359 in control C2, 412 in patient P1, 408 in patient P2, and 181 in patient P3) and calculated the proportion of abnormal mitochondria with large vacuoles or disturbed cristae.

### Microscopy.

Cells were seeded on glass coverslips (*n* = 4 per condition), fixed in 4% paraformaldehyde in PBS for 10 minutes at room temperature, then washed 3 times with PBS and permeabilized in 0.1% Triton X-100 in PBS containing 5% FBS (PBSF) for 30 minutes at room temperature, followed by washing 3 times with PBS. Primary anti-DNA mouse IgM antibodies (PROGEN, catalog 61014) were diluted 1 μg/mL in PBSF. Anti-ATP synthase rabbit Ig antibodies (ATP5A, catalog ab121229, Abcam) were diluted 1/500 in PBSF and incubated with cells overnight at 4°C. After washing 3 times with PBSF, cells were incubated for 1 hour at room temperature with the fluorescent secondary goat anti-mouse IgG–Alexa Fluor 488 antibody (Molecular Probes, catalog A32723) and donkey anti-rabbit IgG–Alexa Fluor 594 antibody (Molecular Probes, catalog A32754), diluted 1/1000 in PBSF. Cells were rinsed 3 times with PBS for 10 minutes, and coverslips were mounted using Fluorescence Mounting Medium (Dako).

Images were acquired using an LSM 700 LIVE DUO confocal microscope (Carl Zeiss Microscopy). For all imaging, *Z*-stack images (1024 × 1024 pixels) were acquired and processed with Bitplane Imaris 4.0 software. Two pictures with at least 8 cells per pictures were analyzed for each line. To measure colocalization in the whole cell, images were processed using Imaris 4.0 (Bitplane) software using the automatic thresholding feature for colocalization. Nucleoid fluorescence area and volume were obtained after maximal projection of images using MetaMorph 5.0 software (Molecular Devices).

### Protein production, crystal structure analysis, and in silico mutants.

Protein production was as previously reported (36). Glycerol was removed from protein samples by gel filtration, and eluted fractions were incubated with 35 nt polycytosine ssDNA purification (4:2.2 Prot:DNA ratio), and dialyzed in 2 steps to 50 mM NaCl, 20 mM TrisHCl pH 7. The final mix was concentrated to 8.6 mg/ml and used for extensive crystallization condition screening. Crystallization condition optimization by the vapor diffusion method at 20°C in 24-well plates sitting drop format (Hampton Research) yielded crystals in 11% PEG1500, 0.1 M cacodylate pH 6.5, 0.2 M magnesium chloride as a reservoir solution. Crystals were incubated in 20% PEG200 and flash frozen in liquid nitrogen. Diffraction data were collected at ALBA Synchrotron Beamline XALOC (Cerdanyola del Vallès, Spain) in collaboration with ALBA staff and integrated, scaled, merged, and reduced with XDS, XSCAL, and XDSCONV (37) at 2.1 Å resolution. A set of 776 (5.2%) reflections was kept aside for Rfree crossvalidation of the model (38). The SSBP1 crystal belonged to the P4_1_2_1_2 tetragonal space group and contained a dimer in the asymmetric unit. Previous human SSBP1 crystal structure (PDB code 3ULL; ref. 15) mutated to polyalanines was used for molecular replacement searches with MOLREP (39). Iterative cycles of manual model building followed with Coot (40) combined with automatic refinement with Phenix (41) and Buster (42) and included TLS refinement and noncrystallographic symmetry. Stereochemistry validation was performed with MolProbity (43), as implemented in the Protein Data Bank. Our sequence assignment clearly indicated a shift compared with the 3ULL structure, from Asn123 to the C terminus. Indeed, 3ULL had previously been revised by the same authors using a structural energy score validation method and taking the *E*. *coli* structure as a reference (PDB code 1S3O) (14), which revealed the sequence shift we experimentally found. However, the new sequence assignment revision did not improve the crystallographic phases, and the electron density was still ambiguous at the C-terminus (14). Our structure, of higher resolution, unambiguously showed the actual sequence of h-mtSSB and confirmed 1S3O. Two β-hairpins (aa 67–73 in molecule A and 66–79 in B) could not be fully traced due to high flexibility. An additional short segment of 4 residues (chain C, not traced in former structures) was sandwiched between β-sheets of symmetry partners, but its side chains were not built due to weak density; its close proximity to the first traced residue in molA suggested it may correspond to the N-terminal end. Stereochemistry validation indicated 100% of traced residues were in allowed Ramachandran regions. Twenty models for the R38Q and R107Q mutants and WT controls were automatically generated with the single mutant routine implemented in Modeller (44). Since arginine and glutamine perform electrostatic interactions, we favored these by setting the electrostatic restraints shell to 15 Å, so that optimization of side chain orientation was not based only in stereochemical clashes. The SSBP1 crystal structure was deposited at the Protein Data Bank (PDB 6RUP). Crystal data and structure refinement are presented in Supplemental Table 1.

### EdU labeling and detection.

For EdU labeling (Invitrogen) to detect and quantify mtDNA synthesis, primary patient and control fibroblasts were grown on coverslips in DMEM (Lonza BE12-604F) supplemented with 10% FCS (GE Healthcare) in a humidified 37°C incubator at 5% CO_2_. All cell lines were routinely tested for mycoplasma contamination and found to be negative. EdU labeling and detection were done as previously described (45) using 100 μM EdU for a 1-hour labeling period and Alexa Fluor 488 azide for detection. Following EdU labeling, cells were incubated with an SSB1 antibody (MilliporeSigma, HPA002866, 1:100) detected with anti-rabbit Alexa Fluor 647 and a DNA antibody (Progen, catalog 61014, mouse monoclonal, IgM, 1:400) detected with an anti-mouse IgM Alexa Fluor 568 antibody. For quantification, EdU and mtDNA foci were manually counted for 3 individual images for each patient cell line and control cell line, as described previously (45).

### Western blot analyses.

Levels of proteins were detected by immunoblot using commercially available antibodies, revealed using chemiluminescence. Pellets were mixed with 100 μl of RIPA lysis buffer (MilliporeSigma) with 1× Protease Inhibitor Cocktail (Roche). The cellular protein content was determined with the BCA Kit (Thermo Fisher Scientific), and 30 μg of total fibroblasts protein was mixed with 2× Laemmli’s sample buffer (Bio-Rad) containing 1/20 dilution of b-mercaptoethanol (MilliporeSigma). The samples were heated 5 minutes at 95°C and loaded onto a 10% polyacrylamide precast MiniProtean TGX gel. The separated proteins were electrotransferred using a Trans-BlotVR TurboTM PVDF Transfer Pack and System. Membranes were saturated with 5% nonfat milk dissolved in 0.1% Tween-TBS for 2 hours at room temperature, then incubated overnight at 4°C with polyclonal sheep anti-SSBP1 (1 μg/ml, catalog AF6588, Bio-techne), monoclonal mouse anti-Grim19 (1:1000, catalog ab110240, Abcam), anti-SDHA (1:1000, catalog ab14715, Abcam), anti-UQCRCII (1:1000, catalog ab14745, Abcam), anti-MTCO1 (1:1000, catalog ab14705, Abcam), anti-GAPDH (1:2000, catalog G8795, MilliporeSigma), and polyclonal rabbit anti-ATP synthase (1:1000, catalog ab151229, Abcam).

Membranes were washed 3 times in 0.1% Tween-TBS and incubated with anti-sheep IgG horseradish peroxidase–conjugated antibody (1:1000, HAF016, Bio-Techne), anti-rabbit IgG, or anti-mouse IgG HRP-linked antibody (1:10 000, AP182P, AP192P, MilliporeSigma) for 2 hours at room temperature. The immunoreactive proteins were visualized with enhanced chemiluminescence (ECL+ Western Blotting Detection Reagents, Amersham Biosciences). Band intensities were quantified with ImageJ (NIH) (*n* = 3 independent experiments).

For sensitivity of mutant and WT SSBP1 to reducing conditions, 3 biological repeats of the 3 patient fibroblast cell lines as well as 2 control fibroblast lines were collected and lysed for 10 minutes on ice in lysis buffer (50 mM Tris-HCl pH 7.4, 150 mM NaCl, 1 mM EDTA, 1% Triton X-100, and 2.5 mM PMSF), followed by a centrifugation step of 14,000 *g* for 5 minutes at 4°C. 60 μg of cellular lysates were separated by SDS-PAGE using sample buffer that included freshly added β-mercaptoethanol to obtain a final concentration in the sample of 1%. SDS-PAGE was followed by Western blotting onto supported nitrocellulose membranes. Membranes were probed with antibodies against proteins of interest and HRP-conjugated secondary antibodies followed by ECL detection. ECL reactions were visualized with a ChemiDoc instrument (Bio-Rad). Antibodies used for Western blot detection were SSBP1 (MilliporeSigma, catalog HPA002866), TFAM (gift of R. Wiesner), POLG1 (Santa Cruz Biotechnology Inc., catalog sc5931), GRSF1 (MilliporeSigma, catalog HPA036985), and actin (Novusbio, catalog NB600-532H). Band intensities were determined and corrected for loading based on the actin signal. The results for the 3 biological repeats of both control cell lines were pooled to obtain single control values, while patient cell lines were treated separately.

### mtDNA deletion analysis.

For Long-Range PCR (LR), control and patient DNA samples (50 ng/μl) were prepared. mtDNA was amplified using 3 pairs of primers: LR1: F1:8285-8314 and R1: 15600-15574 (WT mtDNA fragment of 7315 bp), LR2: F2: 3485-3519 and R2: 14820-14786 (WT mtDNA fragment of 11335 bp), and LR3: F3: 5459-5493 and R3: 735-701 (WT mtDNA fragment of 11845 bp). The PCR conditions were as follows: 1 cycle at 94°C for 1 minute; 30 cycles at 98°C for 10 seconds and 68°C for 11 minutes; and a final extension cycle at 72°C for 10 minutes. PCR was performed using TaKara LA Taq DNA polymerase for the first pair of primers, and TaKara Ex Taq DNA polymerase for the other 2 sets of primers (TaKara Shuzo Corp.).

### DNA extraction.

Total DNA was extracted from the peripheral blood (*n* = 35) using phenol chloroform standard procedures and from the urine (*n* = 29) using the High Pure PCR Template Preparation Kit (Roche).

### mtDNA sequencing and analysis.

The entire mtDNA molecule was amplified as 2 overlapping 8.5 kb fragments. Library preparation was performed using the Ion Plus Fragment Library Kit (catalog 4471269).

Sample emulsion PCR, emulsion breaking, and enrichment were performed using the Ion 540 Kit–Chef (catalog A27759) and mtDNA was sequenced on the Ion S5 Sequencer.

Sequencing database calling and mapping were performed using Ion Torrent Suite. Variant analysis was done with a dedicated in-house bioinformatic pipeline, including the calling, annotation, and prioritization steps. The calling module integrated the prediction of 6 callers. All of the variant calling formats (VCFs) generated were normalized and decomposed before launching the annotation-prioritization module, which combined the ANNOVAR for variant prioritization. ANNOVAR allowed the inclusion of several databases, i.e., Mitomap and MitImpact2, and prioritization tools, i.e., Polyphen2, SIFT, and MutationTaster. Searching for mtDNA deletions and insertions was performed using the eKLIPSE program, which is based on a soft-clipping analysis (18).

### Statistics.

Statistical analyses of the data were carried out using GraphPad Prism software, version 5.00. For analysis of mutation and deletion rates in blood and urine mtDNA, a comparison between both groups was carried out using the nonparametric test (Mann-Whitney *U* test). One-way ANOVA with Dunnett’s correction was used for quantification of mtDNA synthesis and SSBP1 monomer and dimer and to compare the fibroblasts from SSBP1 patients and controls. A *P* value of less than 0.05 was considered significant.

### Study approval.

Informed consent was obtained for clinical examination and genetic analysis from all patients. Informed consent was obtained from patients V:11, VI:17, V:9, and healthy control individuals V:14 and VI:18 for skin biopsy. All methods were carried out in accordance with protocols approved by the Montpellier University Hospital and Tübingen University Hospital and in agreement with the Declaration of Helsinki. The Ministry of Public Health (Paris, France) accorded approval for biomedical research under authorization number 11018S.

## Author contributions

CPM and ES performed the majority of experiments, analyzed data, generated the figures, helped to write the manuscript, and edited the manuscript. The authorship order among co–first authors was determined alphabetically. CD, ES, JNS, and MS developed the concept for the study and designed the experiments. CPM, ES, GM, AS, and BB generated, analyzed, and interpreted the genetic data. HW, UK, NW, BW, AR, CH, and XZ recruited families, collected biosamples, and compiled the clinical data from patients. ATS, AC, and MS generated the crystal structure of SSB1. CPM, ES, CC, MP, FH, MQ, MC, and DG performed experiments and analyzed and interpreted experimental data. AM and GL designed some of the research studies. CD, ES, GL, JNS, and MS wrote the manuscript.

## Supplementary Material

Supplemental data

## Figures and Tables

**Table 1:**
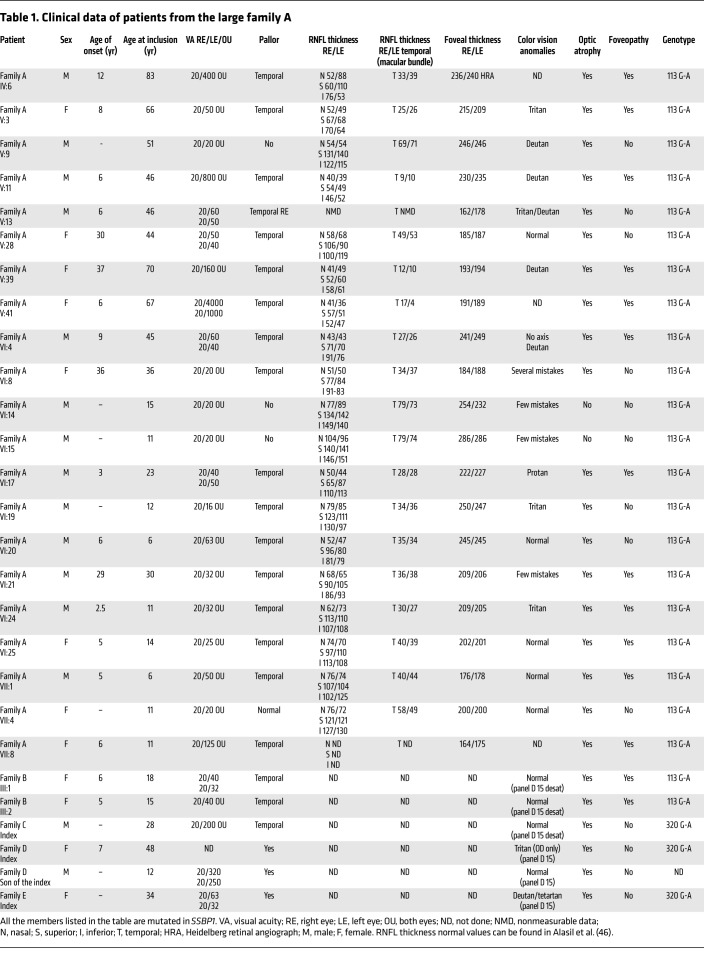
Clinical data of patients from the large family A

## Version 1 09/24/2019

In-Press Preview

## Version 2 11/18/2019

Electronic publication

## Version 3 01/02/2020

Print issue publication
