# Supplementary material for: Dominant mutations in mtDNA maintenance gene SSBP1 cause optic atrophy and foveopathy
Source: J Clin Invest. 2019 Nov 18;130(1):143–56. doi: 10.1172/JCI128513 (PMC6934222; doi:10.1172/JCI128513)
Supplement: Supplemental data [file jci-130-128513-s395.pdf]

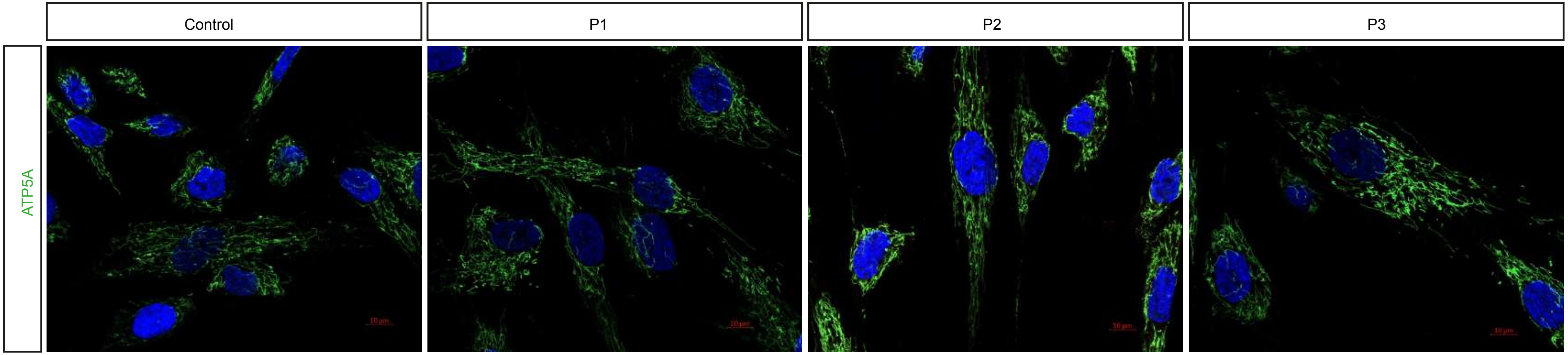
Control
P1
P2
P3
ATP5A

Supplemental figure 1. : Mitochondrial network in primary culture of patient skin fibroblasts. Mitochondrial network was labelled using an anti-ATPsynthase 5A antibody (green). Hoechst was added to label nuclei (blue). P1: Patient 1, P2: patient 2 and P3: patient 3. Scale bar = 10 µm.

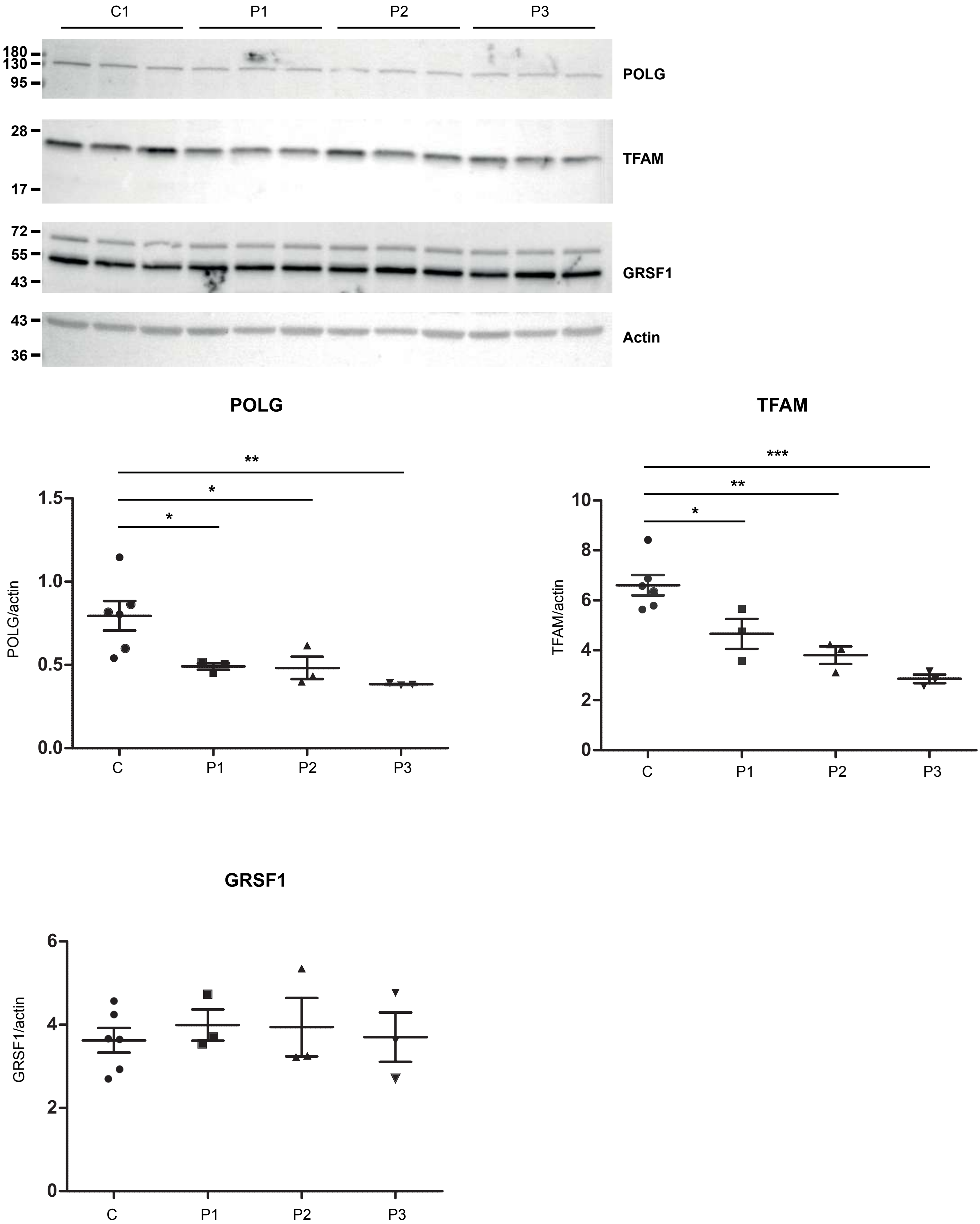

C1
P1
P2
P3
180
130
95
POLG
28
17
TFAM
72
55
43
GRSF1
43
36
Actin
POLG
**
*
*
1.5
1.0
0.5
0.0
POLG/actin
C
P1
P2
P3
TFAM
***
**
*
10
8
6
4
2
0
TFAM/actin
C
P1
P2
P3
GRSF1
6
4
2
0
GRSF1/actin
C
P1
P2
P3

Supplemental figure 2: Analysis of gene involved in mtDNA replication. Western blot of protein responsible for mtDNA replication: POLG, TFAM, GRSF1, with beta-actin as internal control in whole cells lysates from fibroblasts. Densitometric analysis in both controls (C) and patient fibroblasts (P1, P2, P3) are represented at the bottom (mean ± SEM). All data are representative of 3 independent experiments. One way ANOVA with Dunnett's correction was used.

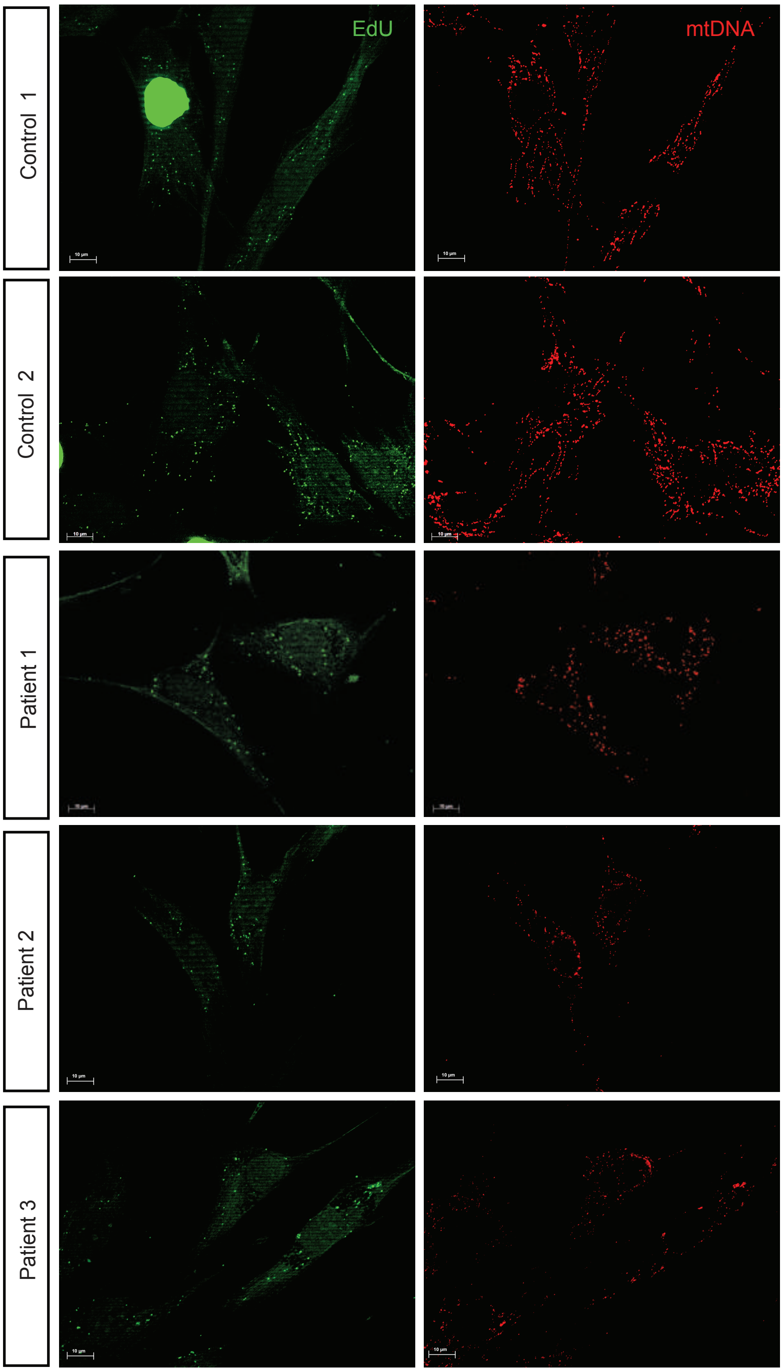
EdU
mtDNA
Control 1
Control 2
Patient 1
Patient 2
Patient 3

Supplemental figure 3. Analysis of mtDNA replication. Assessment of mtDNA replication efficiency on cultured skin fibroblasts from Controls (Control 1 and Control 2) and patients, Patient 1, Patient 2 and Patient 3. 5-ethynyl-2'-deoxyuridine (EdU) labelling was used to detect and quantify mtDNA synthesis while anti-DNA antibody was used to quantify all visible mtDNA foci. Scale bar= 10 µm. Images representing control 2 and patient 1 correspond to control and patient 1 images in Figure 5D.

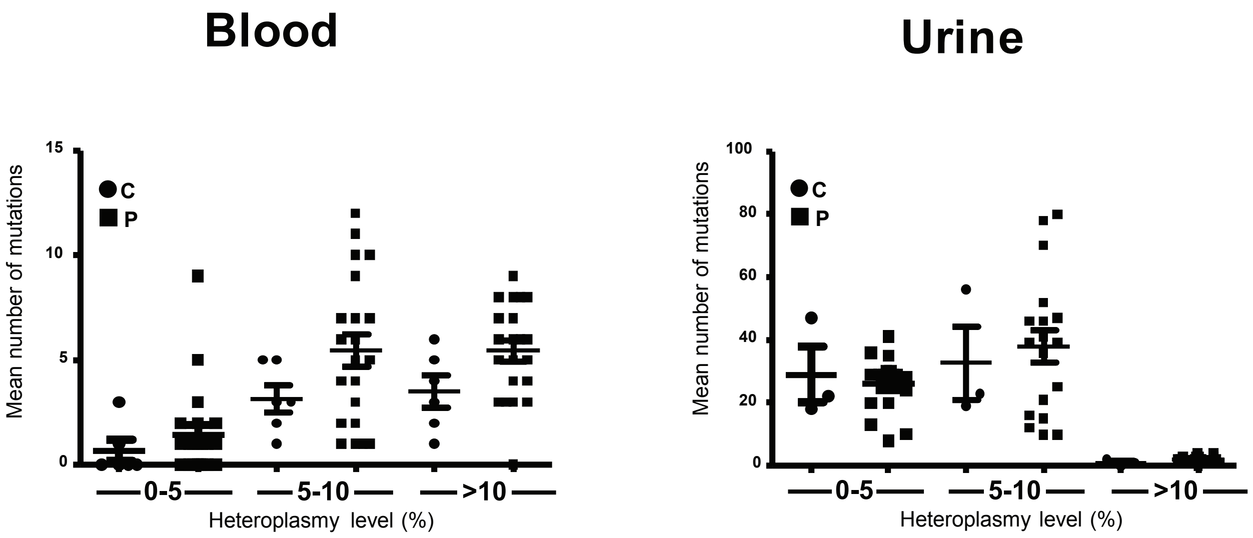

Blood
Urine
Mean number of mutations
C
P
15
10
5
0
100
80
60
40
20
0-5
5-10
>10
Heteroplasmy level (%)

Supplemental figure 4. Mutation frequencies in mtDNA. Mutation frequencies in mtDNA in control (C) and patients in blood (P) and urine. Comparison between the mean number of mutations according to their heteroplasmy level in the mtDNA from controls (C) and patients (P).

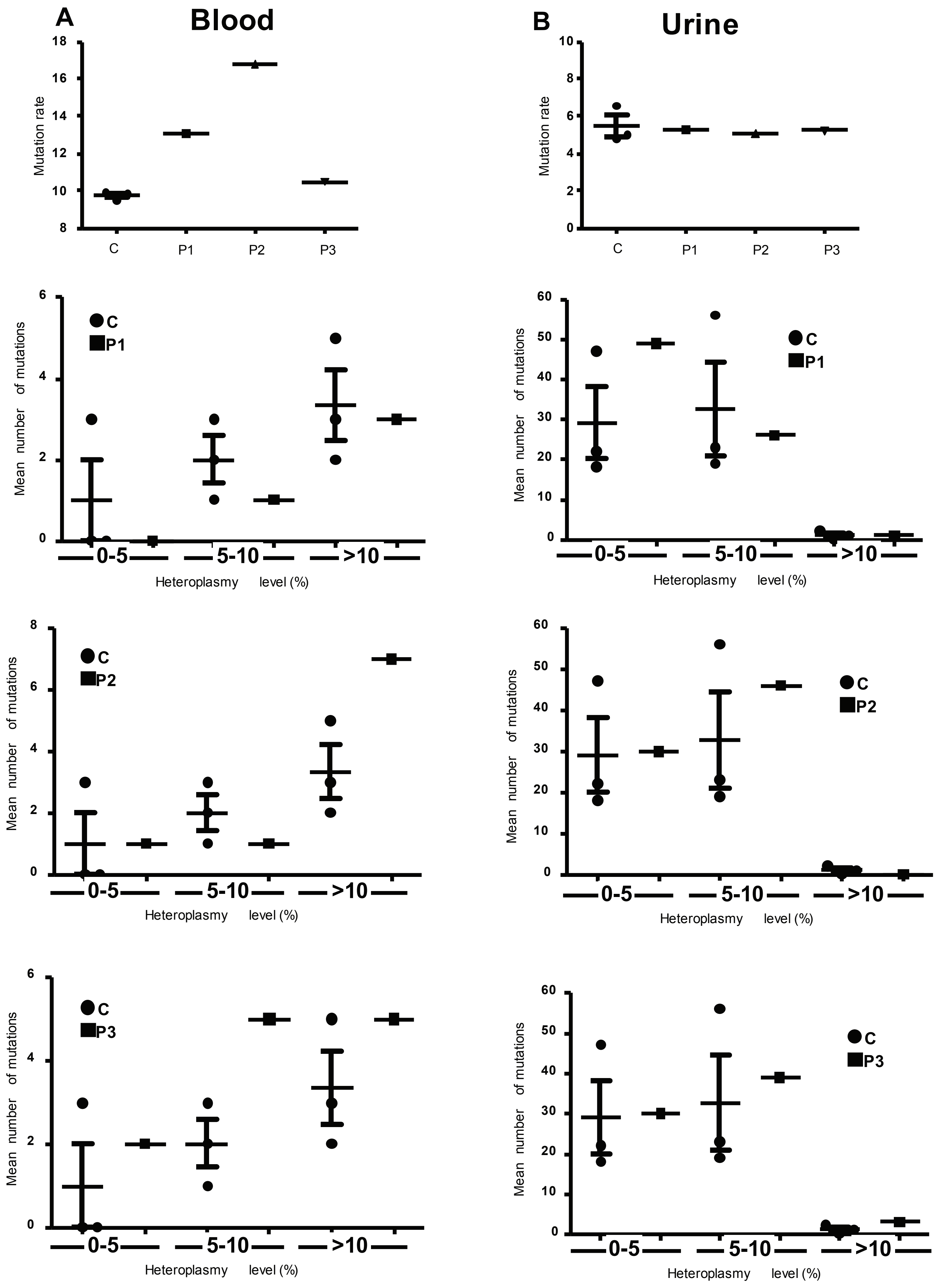

A
Blood
B
Urine
Mutation rate
C
P1
P2
P3
Mean number of mutations
Heteroplasmy level (%)
0-5
5-10
>10

Supplemental figure 5. Mutation frequencies in mtDNA in patient 1, patient 2 and patient 3. **(A)** Mutation rates in mtDNA and frequencies in controls (C) and patients P1, P2, P3 in blood. Top: frequency of new mtDNA mutations identified in blood. Bottom: comparison between the mean number of mutations according to their heteroplasmy level in the mtDNA from controls (C) and patients P1, P2, P3. **(B)** Mutation rates in mtDNA and frequencies in controls (C) and patients P1, P2, P3 in urine. Top: frequency of new mtDNA mutations identified in urine. Bottom: comparison between the mean number of mutations according to their heteroplasmy level in the mtDNA from controls (C) and patients P1, P2, P3.

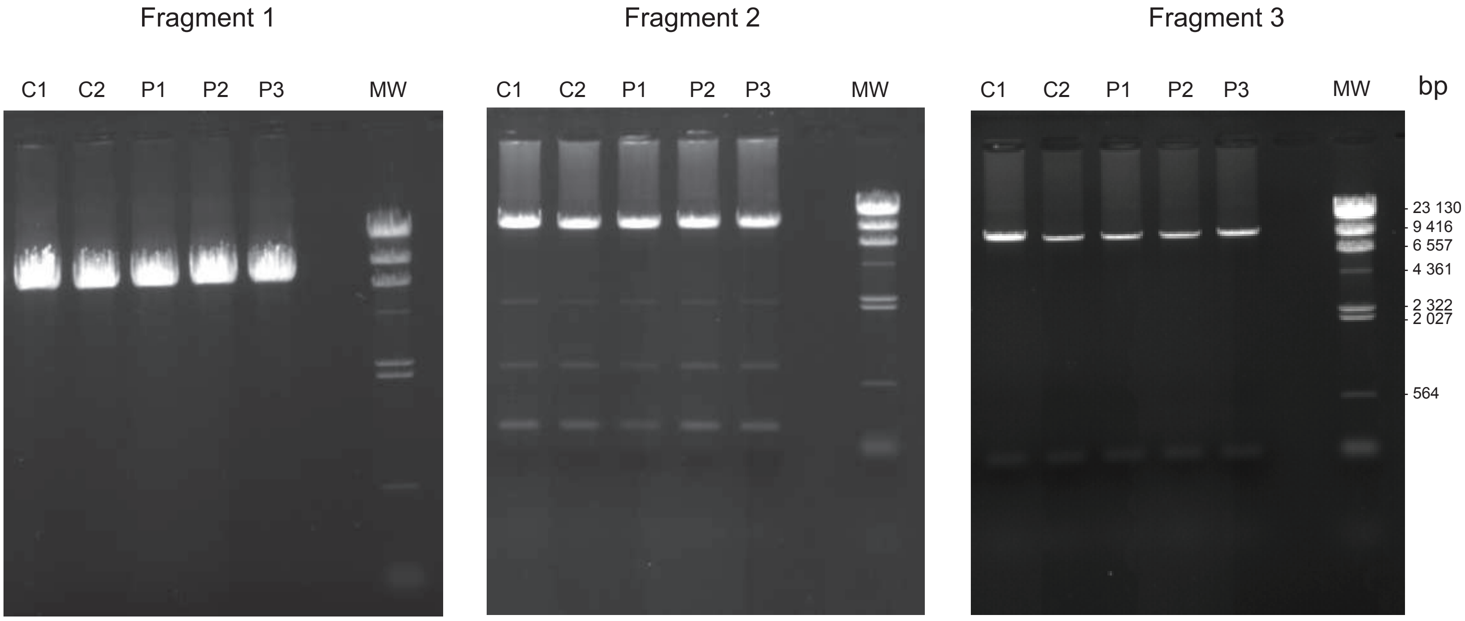

Fragment 1
Fragment 2
Fragment 3
C1
C2
P1
P2
P3
MW
bp
23 130
9 416
6 557
4 361
2 322
2 027
564

Supplemental figure 6: Long-range mtDNA PCR. Mitochondrial DNA was amplified in three distinct PCRs in control fibroblasts (C1, C2) and patient fibroblasts (P1, P2, P3). Fragment 1: 7315 bp, fragment 2: 11.335 bp and fragment 3: 11.845 bp. MW : molecular weight.

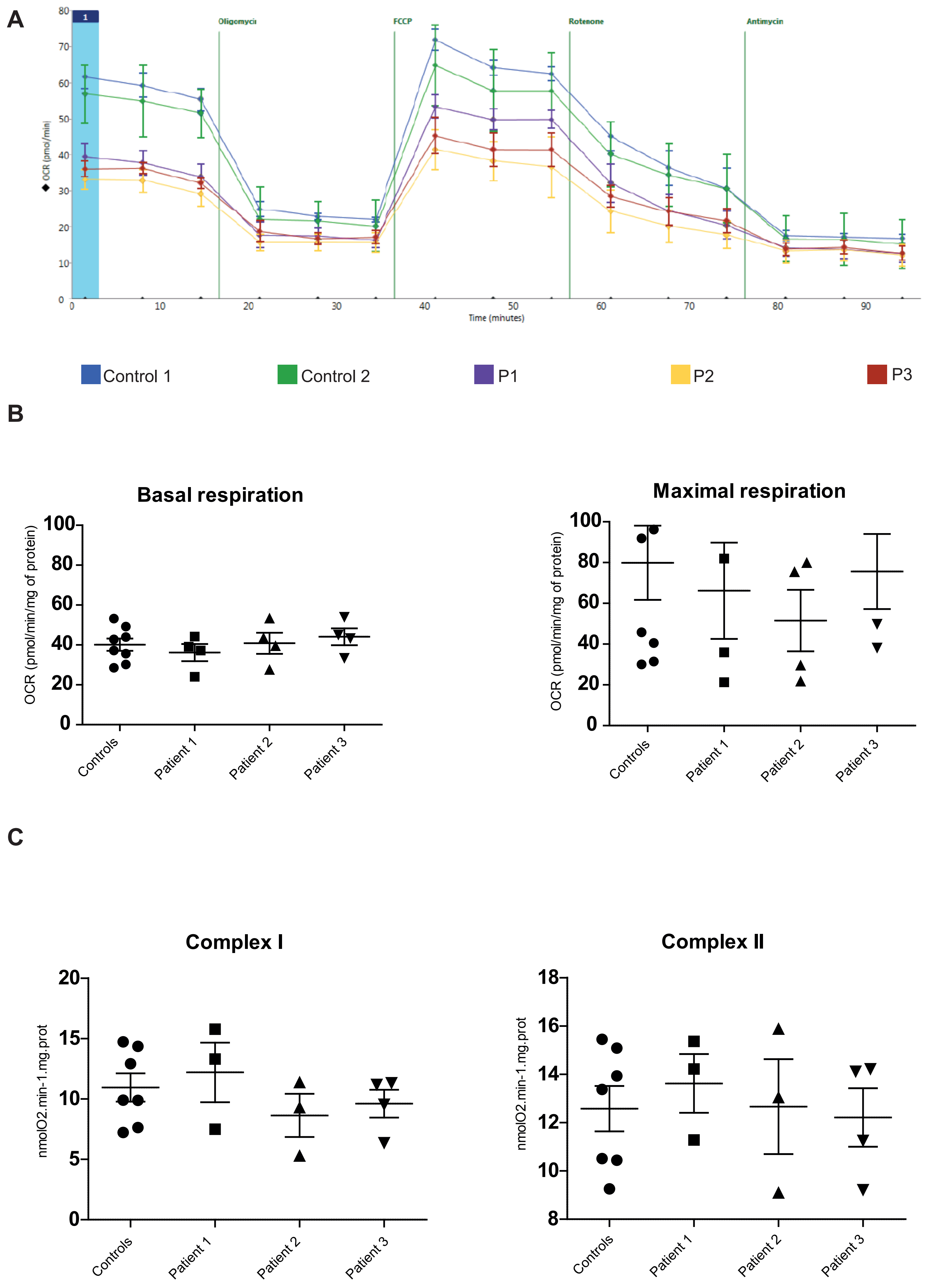
A
Oligomycin
FCCP
Rotenone
Antimycin
OCR (pmol/min)
Time (minutes)
Control 1
Control 2
P1
P2
P3
B
Basal respiration
OCR (pmol/min/mg of protein)
Controls
Patient 1
Patient 2
Patient 3
Maximal respiration
OCR (pmol/min/mg of protein)
Controls
Patient 1
Patient 2
Patient 3
C
Complex I
nmolO2.min-1.mg.prot
Controls
Patient 1
Patient 2
Patient 3
Complex II
nmolO2.min-1.mg.prot
Controls
Patient 1
Patient 2
Patient 3

Supplemental figure 7: Analysis of mitochondrial respiration in control and patient fibroblasts. (**A**) Oxygen consumption rate profiles of both controls (Control 1, Control 2) and patient fibroblasts (P1, P2, P3) using a Seahorse XF96 Extracellular Flux Analyser. (**B**) Oxygen consumption rate (OCR) normalized to protein content for basal respiration (**C**) Oxygen consumption rate (OCR) normalized to protein content for maximal respiration. (**D**) Mitochondrial complex I- dependent respiratory rate in controls (Controls) and patient fibroblasts (Patient 1, Patient 2, Patient 3). (**E**) Mitochondrial complex II- dependent respiratory rate in controls (Contols) and patient fibroblasts (Patient 1, Patient 2, Patient 3). Respiratory rate: nmol oxygen consumed/min/mg protein. (mean ± SEM).

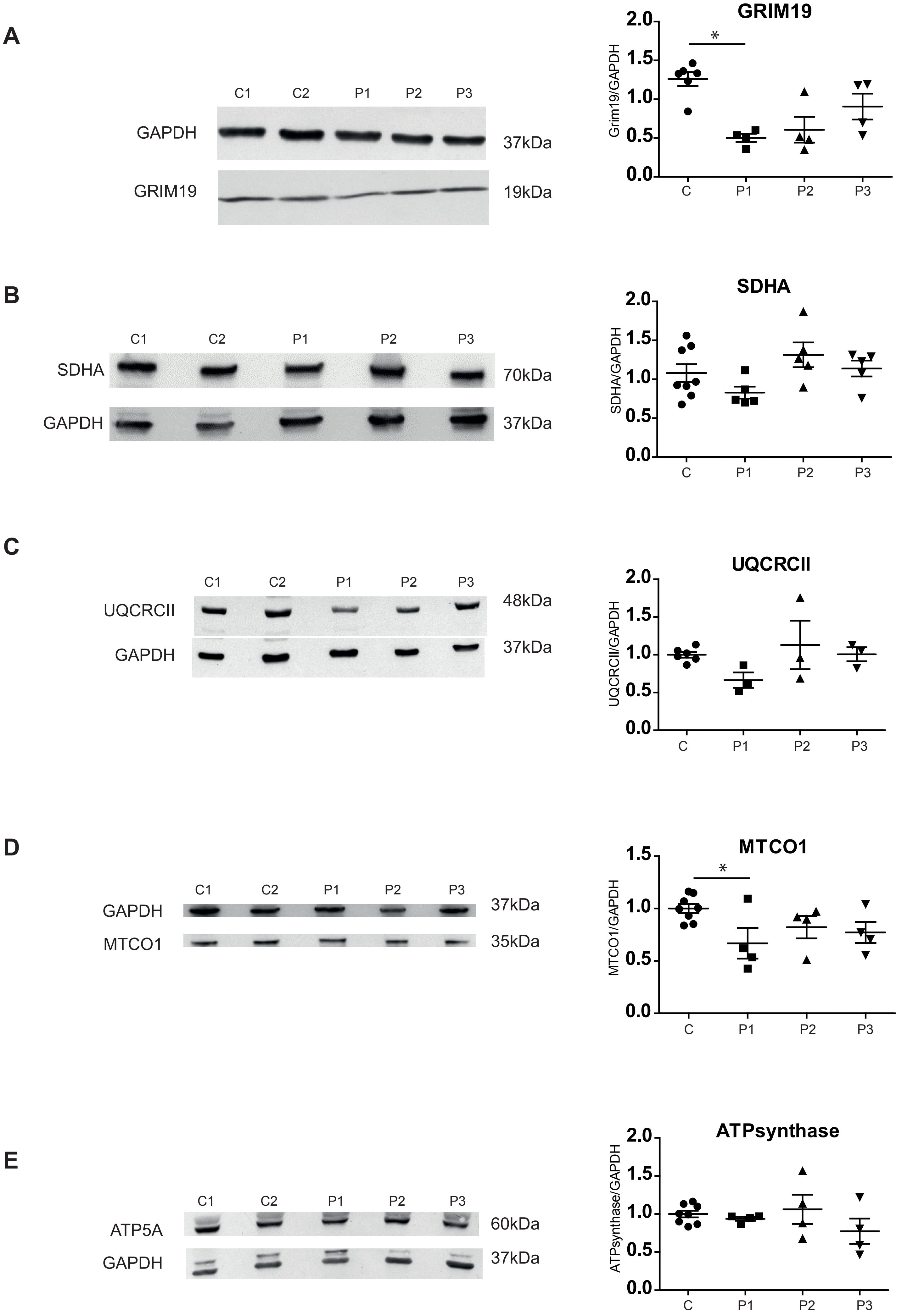

A
C1 C2 P1 P2 P3
GAPDH
37kDa
GRIM19
19kDa
GRIM19
Grim19/GAPDH
2.0
1.5
1.0
0.5
0.0
*
C P1 P2 P3
B
C1 C2 P1 P2 P3
SDHA
70kDa
GAPDH
37kDa
SDHA
SDHA/GAPDH
2.0
1.5
1.0
0.5
0.0
C P1 P2 P3
C
C1 C2 P1 P2 P3
UQCRCII
48kDa
GAPDH
37kDa
UQCRCII
UQCRCII/GAPDH
2.0
1.5
1.0
0.5
0.0
C P1 P2 P3
D
C1 C2 P1 P2 P3
GAPDH
37kDa
MTCO1
35kDa
MTCO1
MTCO1/GAPDH
1.5
1.0
0.5
0.0
*
C P1 P2 P3
E
C1 C2 P1 P2 P3
ATP5A
60kDa
GAPDH
37kDa
ATPsynthase
ATPsynthase/GAPDH
2.0
1.5
1.0
0.5
0.0
C P1 P2 P3

Supplemental figure 8: Expression of mitochondrial respiratory chain proteins.

Western blot of (**A)** GRIM19 (complex I), (**B)** SDHA (complex II), (**C)** UQCRC2 (complex III), (**D)** MTCO1 (complex IV) and (**E)** ATP-synthase (complex V), in pooled control (C) and patient (P1, P2, P3) fibroblasts. GAPDH was used as a loading control. Densitometric analysis in control and patient fibroblasts are represented at the right (mean ± SEM). *P**<0.05. One way ANOVA with Dunnett's correction was used.

Supplementary Table 1. Crystal data and structure refinement

**Data collection**	
Wavelength (Å)	0.979500
Resolution range (last shell) (Å)	45.58 – 2.10 (2.23-2.10)
Space group	$P4_12_12$
Unit Cell (a,b,c, α, β, γ)	51.04, 51.04, 182.34, 90.0, 90.0, 90.0
Number of unique reflections measured	14943 (2342)
Multiplicity	11 (11.2)
Completeness (%)	99.5 (97.5)
Mean I/sigma (I)	16.28 (1.09)
Wilson B-factor (Å)	56.15
R-factor (%)	9.6 (245.8)
Rmerge (%)	9.1 (234.6)
CC1/2	99.9 (55.1)
**Refinement**	
Resolution range	45.58 – 2.10
Total reflections used in refinement	14874
Reflections used for R-free (% from total)	776 (5.22)
R-work (%)/ R-free (%)	20.0/ 24.9
Protein atoms	1764
Solvent atoms	95
Mg^{2+} atoms	2
RMS deviation bonds (Å)	0.008
RMS deviation angles (°)	0.98
Mean B-factor ($Å^2$)	72.12
Ramachandran most favoured (%)	94
Ramachandran additional allowed (%)	6

Supplementary Table 1. Crystal data and structure refinement

In brackets, values in last shell. Rfactor=$\sum|(I_{h,i}-I_h)|/\sum |I_{h,i}|$. R-meas=redundancy independent R-factor of intensities (1) Rwork =$\sum|(F_o-F_c)|/\sum |F_o|$ for the working set of reflections, Rfree is the same for the test set (2)

1. Diederichs, K., and Karplus, P.A. 1997. Improved R-factors for diffraction data analysis in macromolecular crystallography. *Nat Struct Biol* 4:269-275.
2. Brunger, A.T. 1992. Free R value: a novel statistical quantity for assessing the accuracy of crystal structures. *Nature* 355:472-475.
